# Serum Metabolomics to Identify the Liver Disease-Specific Biomarkers for the Progression of Hepatitis to Hepatocellular Carcinoma

**DOI:** 10.1038/srep18175

**Published:** 2015-12-10

**Authors:** Rong Gao, Jianhua Cheng, Chunlei Fan, Xiaofeng Shi, Yuan Cao, Bo Sun, Huiguo Ding, Chengjin Hu, Fangting Dong, Xianzhong Yan

**Affiliations:** 1 National Center of Biomedical Analysis, Beijing 100850, China; 2 Department of Gastroenterology and Hepatology, Beijing You’an Hospital affiliated to Capital Medical University, Beijing 100069, China; 3 Key Laboratory of Molecular Biology for Infectious Diseases, Ministry of Education, China; Department of Infectious Diseases, the Second Affiliated Hospital of Chongqing Medical University, Chongqing 400010, China; 4 Department of Laboratory Medicine, the General Hospital of Jinan Military Command, Jinan 250031, Shandong, China

## Abstract

Hepatocellular carcinoma (HCC) is a common malignancy that has region specific etiologies. Unfortunately, 85% of cases of HCC are diagnosed at an advanced stage. Reliable biomarkers for the early diagnosis of HCC are urgently required to reduced mortality and therapeutic expenditure. We established a non-targeted gas chromatography–time of flight–mass spectrometry (GC-TOFMS) metabolomics method in conjunction with Random Forests (RF) analysis based on 201 serum samples from healthy controls (NC), hepatitis B virus (HBV), liver cirrhosis (LC) and HCC patients to explore the metabolic characteristics in the progression of hepatocellular carcinogenesis. Ultimately, 15 metabolites were identified intimately associated with the process. Phenylalanine, malic acid and 5-methoxytryptamine for HBV *vs*. NC, palmitic acid for LC *vs*. HBV, and asparagine and β-glutamate for HCC *vs*. LC were screened as the liver disease-specific potential biomarkers with an excellent discriminant performance. All the metabolic perturbations in these liver diseases are associated with pathways for energy metabolism, macromolecular synthesis, and maintaining the redox balance to protect tumor cells from oxidative stress.

Hepatocellular carcinoma (HCC) is the third leading cause of cancer-related deaths worldwide with rising incidence^1^, especially in developing countries, which account for 84% of the total incidence and 83% of the total deaths^2^. The geographical variation in HCC incidence is closely associated with the global distribution of hepatitis B virus (HBV) and hepatitis C virus (HCV) infection^2^. Patients with chronic liver disease are at the highest risk of developing HCC^3^. According to the Asia Pacific Working Party on Prevention of Hepatocellular Carcinoma, more than two thirds of the people who die of HCC each year live in Asia and almost 55% of them live in China^4^,^5^. Chronic HBV is the most common cause of HCC in China, which may progress to liver cirrhosis (LC), and has dramatically increased the incidence of HCC^6^. The overriding risk factor accounting for 80%–90% of HCC cases, regardless of etiology, is the presence of a preneoplastic cirrhotic liver, which masks the symptoms of cancer progression^3^,^7^. Due to its rapid development and early metastasis, patients with hepatocellular carcinoma have a very dismal 5-year-survival rate and poor prognosis^8^. Therefore, early discrimination of HCC from high-risk populations, such as those with HBV and LC, is an urgent task to achieve better prognosis and longer survival.

Abnormal metabolism is a universal characteristics of cancer cells, such as aerobic glycolysis, which presumably sustains the accumulation of biomass that is necessary for fast cell growth and proliferation in a tumor microenvironment^9^. Despite our increasing understanding of the molecular pathogenesis of HCC, few reliable and robust biomarkers are available in clinical diagnosis. The widely used HCC biomarker alpha-fetoprotein (AFP) is unsatisfactory because of its high false positive and false negative ratio^10^. Therefore, more sensitive and specific markers for HCC are needed.

Rapid development in metabolomics has made it a very useful technology in disease phenotyping and biomarker generation^11^, which has been increasingly applied in clinical fields^12^. Recently, great efforts in metabolomics field have been made in searching for HCC markers,^11^,^13^,^14^,^15^,^16^,^17^ with some metabolites being selected as potential biomarkers. However, few of these studies concerned the whole processes of the development and progression of HCC from viral hepatitis^18^. Additionally, the small sample sizes and high dimensionality of metabolomics data, and the non-linear relationships and interactions of many variables (metabolic components) make it difficult to use the traditional methods for multiple linear regression analysis. Random Forest (RF) is a powerful and scalable machine learning classification algorithm, which has a good tolerance for outliers and noise, can avoid the over-fitting problem and provide a high prediction accuracy compared with the widely used partial least squares discriminant analysis (PLS-DA)^19^.

In this study, we discriminated the serum profiles of healthy controls (NC), HBV, LC and HCC to comprehensively investigate the metabolites associated with hepatocarcinogenesis and identify potential biomarkers of each liver disease status. Initially, two-thirds of each group serum specimens were randomly selected as training sets and the rest as validation sets. To ensure the quality and robustness of our measurements, four quality control sets, including an internal standard, blank samples, “pooled” samples and duplicate samples, were run during the sample sequence. To obtain reliable markers and accurate diagnosis of HBV, LC and HCC, unsupervised principal components analysis (PCA) and supervised projection to latent structure with discriminant analysis (OPLS-DA), Random Forests (RF), the binary logistic regression and the Bayes’ multi-group stepwise discriminant analysis were applied on the training set, and the potential biomarkers were further validated by the validation set. Ultimately, we identified 15 metabolites related to the stepwise hepatocarcinogenesis, uncovered robust and technically validated potential biomarkers of HBV, LC and HCC and established a reliable Bayes’ multi-group stepwise discriminant model that can aid clinical diagnosis and guide therapeutic decisions.

## Results

Forty-nine HBV patients, 52 LC patients, 39 HCC patients and 61 healthy subjects were enrolled. The metabolic profiling of serum samples was performed by using the GC-TOFMS in a random order and the representative total ion current (TIC) chromatograms of NC, HBV, LC and HCC are shown in Supplementary Figure S1.

### Metabolic profiling of the samples

**Figure 1:**
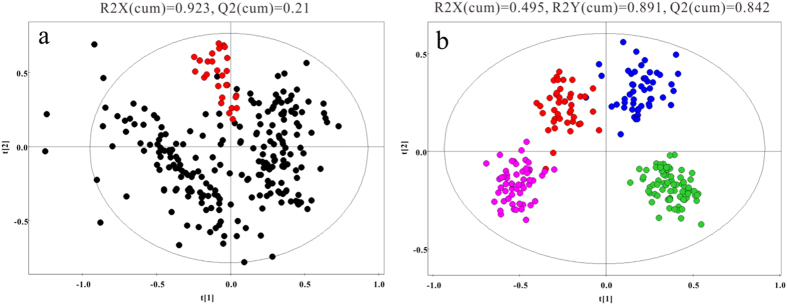
The metabolic profiles of serum samples. (**a**), PCA score plot for the QCs (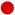) and the other samples (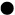), showing that the QC samples cluster together. (**b**), the OPLS-DA score plot for NC (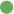), HBV (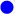), LC (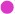) and HCC (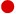), showing an obvious separation between four groups.

After alignment and normalization of the data sets, multivariate statistical analyses were conducted. The PCA scores plot (Fig. 1a) shows a clear cluster of the QC samples (R2X = 0.923, Q2 = 0.21), indicating the high stability and reproducibility of the instrument. The TIC of the blank sample indicate no sample carryover (See Supplementary Figure S1). Additionally, the reproducibility was assessed by the Pearson correlation coefficient of the 30 randomly selected duplicate samples. The high correlation coefficients (≥0. 97, See Supplementary Table S1) indicate that the analysis run had satisfactory repeatability and chromatogram consistency.

The OPLS-DA model after excluding the outliers observed in the PCA reveal an obvious separation between groups (Fig. 1b). Furthermore, the results of hierarchical cluster analysis (HCA), displayed as dendrograms with heatmap based on the Euclidean distance and the Ward’s method, provide an intuitive visualization of the metabolic remodeling in disease groups compared to the NC (See Supplementary Figure S2). Both the OPLS-DA model and HCA show a clear separation between NC, HBV, LC and HCC, indicating that metabolic alterations occurred during liver disease progression.

### Differential metabolites related to stepwise hepatocarcinogenesis

**Figure 2:**
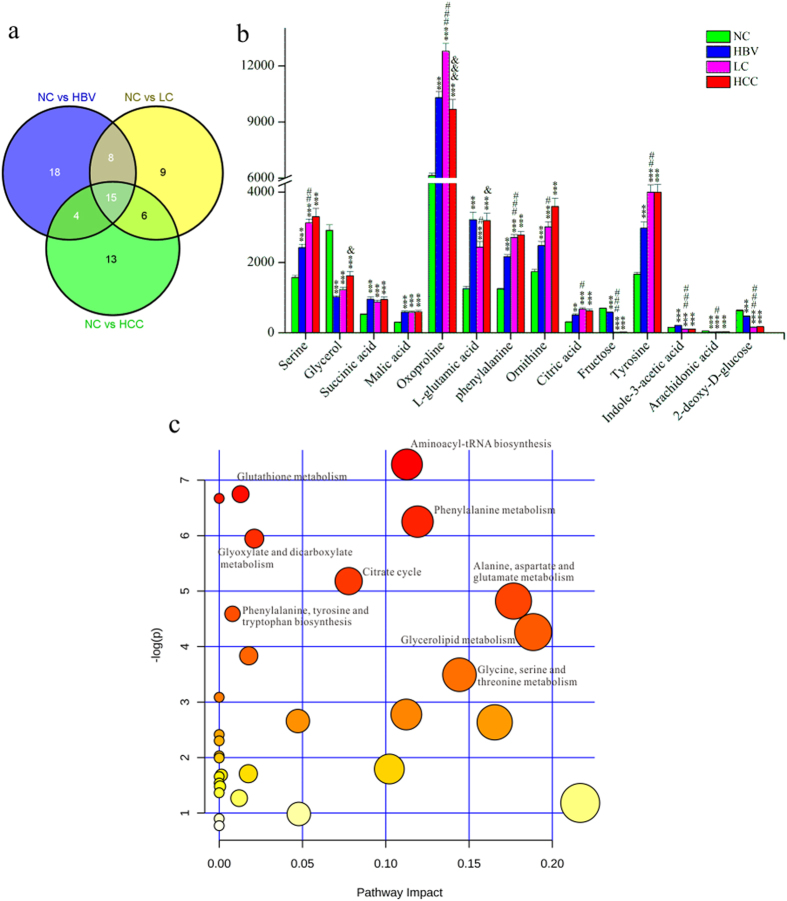
Differential metabolites related to HCC development. (**a**), Venn diagram of the differential metabolites in different liver diseases compared with the healthy controls. (**b**), the relative peak intensity of 15 differentially expressed metabolites in all four groups.*p < 0.05, **p < 0.01 and ***p < 0.001 compared with the NC group, ^#^p < 0.05, ^##^p < 0.01 and ^###^p < 0.001 compared with the HBV group and ^&^p < 0.05, ^&&^p < 0.01 and &&&p < 0.001 compared with the LC group. (**c**), the summary of aberrant pathways in the liver disease group, as analyzed by MetaboAnalyst.

Approximately 80% of HCC develops from liver cirrhosis^14^, which predominantly progresses from HBV in China^20^. We hypothesized that changes in certain metabolites emerge in the early stage of liver diseases and evolve with the disease progression. These metabolites could be considered as associated with the carcinogenesis and HCC development, and might be useful in the screening of high-risk population and in the early diagnosis of HCC. To this end, pair-wise comparisons were carried out based on OPLS-DA models (See Supplementary Figure S3). Ultimately, 45, 38 and 38 significantly changed metabolites with variable importance in the projection values >1.0 and *P* < 0.05 were found in the HBV, LC and HCC groups compared with the NC, respectively (Fig. 2a). Among these metabolites, 15 were consistently altered (Fig. 2a,b). The panel of metabolites includes remarkably elevated serine, succinic acid, malic acid, oxoproline, L-glutamic acid, phenylalanine, ornithine, citric acid, tyrosine, and decreased glycerol, fructose, arachidonic acid, and 2-deoxy-D-glucose in all three groups, whereas indole-3-acetic acid was elevated in HBV, but reduced in LC and HCC.

The biological pathways involved in the metabolism of these 15 metabolites and their biological roles were determined by enrichment analysis using MetaboAnalyst (Fig. 2c). All matched pathways were shown according to *p* values from the pathway enrichment analysis (y-axis) and pathway impact values from pathway topology analysis (x-axis)^21^, with the most impacted pathways colored in red. Consequently, nine pathways were considered closely related to the carcinogenesis and the development of HCC. These includes aminoacyl-tRNA biosynthesis, phenylalanine metabolism, glutathione metabolism, glyoxylate and dicarboxylate metabolism, alanine, aspartate and glutamate metabolism, the citrate cycle, phenylalanine, tyrosine and tryptophan biosynthesis, glycerolipid metabolism, and glycine, serine and threonine metabolism.

### Biomarkers of liver disease status

**Figure 3:**
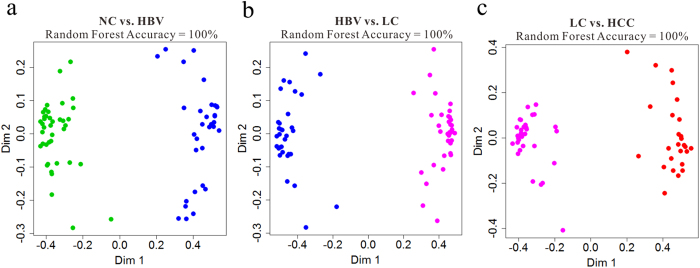
Random Forests (RFs) analyses based on the training data set from NC (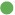), HBV (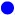), LC (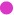) and HCC (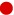). (**a**), Separation by RFs analysis of HBV *vs*. NC; (**b**), Separation by RFs analysis of LC *vs*. HBV; (**c**), Separation by RFs analysis of HCC *vs*. LC.

**Figure 4:**
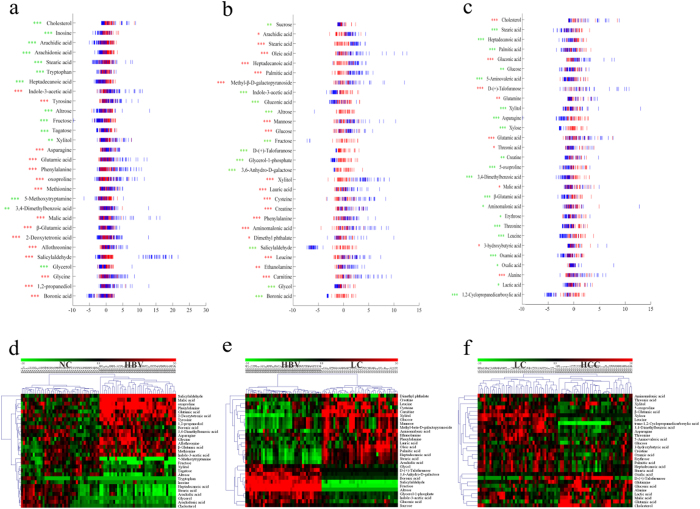
The Z-score plot of the top 30 differentially expressed metabolites based on the RFs analyses in HBV, LC and HCC, relative to their corresponding control groups in the training samples. The values were standardized by the mean values and the standard deviations (SDs) of the corresponding controls in each group. Each vertical line represents one metabolite in one sample. (**a**), Z-score plot for HBV (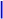) *vs*. NC (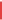); (**b**), Z-score plot for LC (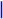) *vs*. HBV (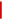); (**c**), Z-score plot for HCC (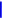) *vs*. LC (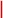). Asterisks indicate the statistical significance between HBV, LC and HCC and their corresponding control groups (green, downregulated; red, upregulated). *p < 0.05, **p < 0.01 and ***p < 0.001. (**d–f**), Heat map of the top 30 differential metabolites in (**d**) HBV *vs*. NC, (**e**) LC *vs*. HBV and (**f**), LC *vs*. HCC.

Further step-wise comparison was also carried out between HBV and NC, LC and HBV, and HCC and LC to explore the metabolic perturbation in these three processes of HCC development. Random Forests, which cope well with high dimensional data, were used to discriminate HBV, LC and HCC from their corresponding control groups, based on the training sets, and yielded excellent classification accuracy of 100% in the three models (Fig. 3a–c). Additionally, the prediction of validation data based on training set RF models using predict function also yielded satisfactory results with classification accuracy of 100% for HBV vs. CN, 100% for LC vs. HBV and 96.77% for HCC vs. LC, demonstrating the goodness of the models. To obtain potential biomarkers for HBV, LC and HCC, the top 30 ranked differential metabolites in the respective models were selected according to the mean decrease accuracy (MDA), which denoted the percent decrease in accuracy when the trial was performed in the absence of the metabolite (Supplementary Figure S4 A–C). Z-score plots of these metabolites in HBV, LC and HCC relative to their corresponding control groups are shown in Fig. 4a–c.

In the serum samples of HBV patients, fatty acids (heptadecanoic acid, stearic acid, arachidonic acid and arachidic acid), carbohydrates (xylitol, tagatose, fructose and altrose), glycerol and cholesterol were significantly downregulated, while the majority of amino acids (glycine, β-glutamic acid, allothreonine, methionine, oxoproline, phenylalanine, glutamic acid, asparagine and tyrosine), malic acid and indole-3-acetic acid were considerably upregulated compared with the NC group. However, the trends were partially reversed in LC *vs*. HBV, where the fatty acids, glucose, xylitol, and mannose were strongly increased, whereas the amino acids were significantly decreased. In the case of HCC *vs*. LC, however, most metabolites were significantly downregulated, while cholesterol, 3-hydroxybutyric acid, malic acid, glutamine, alanine, glutamic acid, gluconic acid, talose and threonic acid were up-regulated. These changes are further visualized in the heatmaps as the HCA results (Fig. 4d–f).

**Figure 5:**
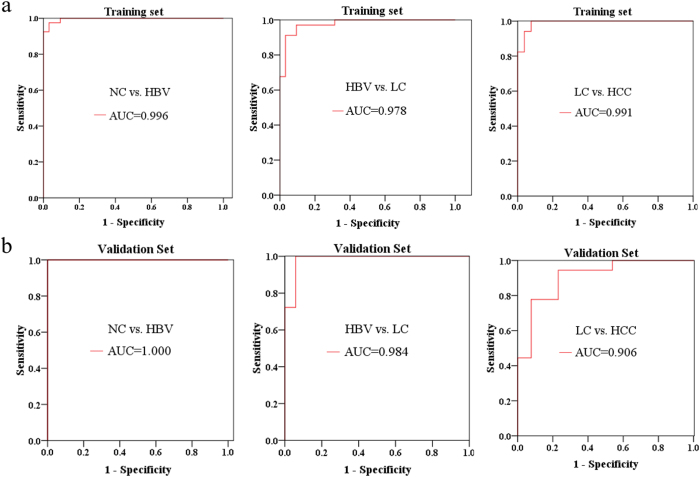
ROC curves, showing the ability of the potential biomarkers to distinguish HBV, LC and HCC from their corresponding control groups, based on (a) the training data set and (b) the validation data set. AUC, area under the curve.

To validate the importance of these metabolites from the Random Forests analysis and to further screen out a group of metabolites as potential biomarkers to accurately stratify subjects into their appropriate groups. Binary logistic regression was then conducted to identify an optimal combination of metabolites as the potential biomarkers for HBV, LC and HCC. Three metabolites (phenylalanine, malic acid and 5-methoxytryptamine) were selected for HBV *vs*. NC, one metabolite (palmitic acid) for LC *vs*. HBV, and two metabolites (asparagine and β-glutamate) for HCC *vs*. LC. ROC curves were then used to evaluate the diagnostic performance of these biomarkers (Fig. 5a,b). The area under the curve (AUC) of the ROC for the training set of HBV *vs*. NC is 0.996, with a sensitivity of 100% and a specificity of 92.5%, while the AUC for the validation set is 1.00, with a sensitivity of 100% and a specificity of 95.2%. For LC *vs*. HBV, the AUC for training set is 0.978, with a sensitivity of 94.1% and a specificity of 90.2%, while the AUC is 0.984 for the validation set, with a sensitivity of 83.3% and a specificity of 100%. The AUC for the training set of HCC *vs*. LC is 0.991, with a sensitivity of 96.2% and specificity of 85.3%, while the AUC for the validation set is 0.906, with a sensitivity of 76.9% and specificity of 83.3%.

### Multiple discriminant analysis of HCC progress

To meet the increasing needs of distinguishing the severity of an unknown patient in clinical practice, a Bayes discriminant function model was established by stepwise discriminant analysis using the training data set. The initial discriminant factors in classification functions included 30 metabolites. By stepwise discriminant analysis, 30 statistically significant variables were entered into the final discriminant function models and retrospective discrimination was conducted among the individuals in the training set (Supplementary Table S2; Supplementary Figure S5). The model achieved an excellent discriminant performance, with a sensitivity of 100%, specificity of 100% and accuracy of 100% (Supplementary Table S3). Subsequently, using the established model, the subjects in the validation set were then discriminated and classified, with correction rates of 100% for NC, 94.12% for HBV, 100% for LC and 76.92% for HCC (Supplementary Table S3).

## Discussion

Reprogramming of cellular metabolism (in particular glycolysis and the TCA cycle) is essential for hepatocarcinogenesis to augment anabolic metabolism to sustain cancer cell growth and proliferation^22^,^23^. In present study, a non-targeted GC-TOFMS metabolomics method was used to explore the metabolic characteristics in the progression of hepatocarcinogenesis and to screen for meaningful and vital liver disease-specific biomarkers for the early and differential diagnosis of hepatopathy, especially for early diagnosis of HCC.

Most HCC cases are developed from liver cirrhosis (LC), which is primarily caused by chronic HBV^24^,^25^. Therefore, we hypothesized that metabolites that changed significantly in HBV, LC and HCC compared with health controls would be intimately associated with the progression of hepatocarcinogenesis. Based on this hypothesis, we identified 15 metabolites that were involved in certain crucial metabolic pathways (Fig. 2c): the aminoacyl-tRNA biosynthesis, phenylalanine metabolism, glutathione metabolism, glyoxylate and dicarboxylate metabolism, alanine, aspartate and glutamate metabolism, citrate cycle, phenylalanine, tyrosine and tryptophan biosynthesis, glycerolipid metabolism and glycine, serine and threonine metabolism.

**Figure 6:**
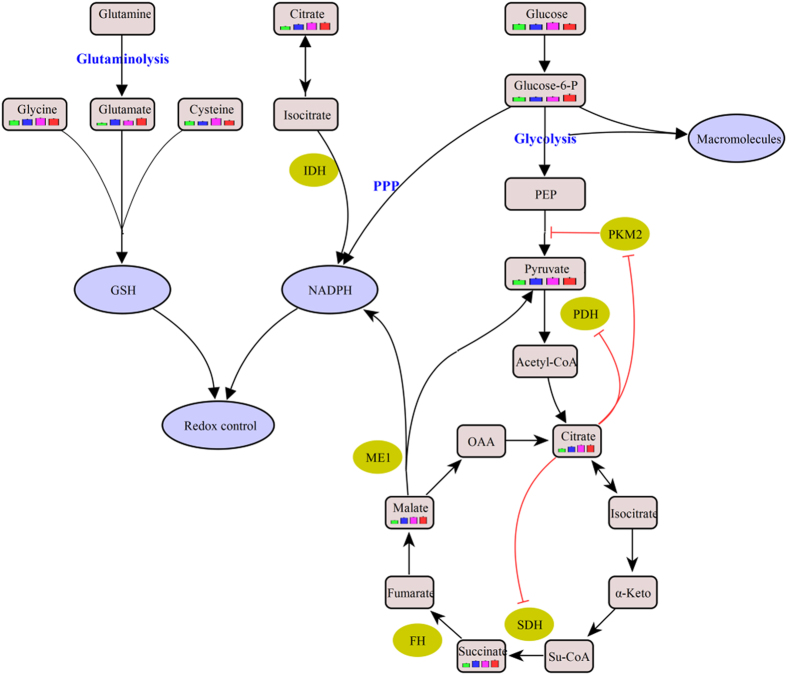
The metabolic pathways related to liver diseases. This schematic shows our current understanding of how glycolysis, the pentose phosphate pathway, oxidative phosphorylation and glutaminolysis are interconnected in cancer cells to form a balance between energy requirements and biosynthesis for cell proliferation.

Interestingly, we observed that three pivotal Krebs cycle intermediates, succinic acid, malic acid and citric acid, were significantly accumulated in the liver disease groups (Fig. 2b, Supplementary Figure S6)^26^. In fact, succinate dehydrogenase (SDH), a key enzyme in the TCA cycle catalyzing the oxidation of succinate to fumarate, also acts as a tumor suppressor. Its deficiency or mutation in some cancers reduces the conversion of succinate to fumarate, resulting in the accumulation of succinate^27^,^28^. Succinate can competitively bind to and inhibit the activity of HIF-1 prolyl dehydrogenase (PHD), a member of the α-ketoglutarate-dependent dioxygenase superfamily, leading to increased stability of HIF-1α and HIF-2α, which are linked to oncogenesis^22^,^29^. HIF-1 can regulate many genes related to tumorigenesis^30^, which stimulates the conversion of glucose to pyruvate by upregulating glucose transporter (GLUT) isoform 1 (GLUT1) and hexokinase (HK)^23^, and then to lactate through lactate dehydrogenase A (LDHA)^30^. Additionally, as an essential metabolic intermediate and a key regulator of energy production, citric acid, which inhibits phosphate fructose kinase 1 (PFK1), pyruvate kinase (PK), pyruvate dehydrogenase (PDH) and succinate dehydrogenase (SDH), was remarkably increased in liver disease groups, consistent with its effect of negative regulation on glycolysis and the TCA cycle (Fig. 6)^31^. Therefore, the elevated malic acid, citric acid and SDH substrate succinic acid observed in this study are likely to be both caused by and to cause the blockade of the TCA cycle, which necessitates a metabolic reliance upon glycolysis, promoting hepatocarcinogenesis (Fig. 6).

In cancer cells, oxidative phosphorylation (OXPHOS) is defected, and acetyl-CoA converted from pyruvate enters a truncated TCA cycle, and is then converted to citric acid, which is preferentially transferred to cytosol where it is converted to acetyl-CoA for cell growth and proliferation^23^,^31^ and oxaloacetate for the production of malic acid, which is reimported into mitochondria^32^. The *de novo* lipid synthesis in tumor cells is enhanced through this mechanism^33^. Furthermore, the major cellular source of reactive oxygen species (ROS) is OXPHOS, whose defects help defense against the damages caused by ROS.

Potential biomarkers of the three stages of HCC development (HBV *vs*. NC, LC *vs*. HBV and HCC *vs*. LC) were revealed by RF analysis combined with the binary logistic regression analysis (Fig. 3, Fig. 5). Finally, phenylalanine, malic acid and 5-methoxytryptamine for HBV *vs*. NC; palmitic acid for LC *vs*. HBV; and asparagine and β-glutamate for HCC *vs*. LC were selected as potential biomarkers. Their AUC values indicated a satisfactory performance in both the training and validation data sets, with remarkable sensitivity and specificity to accurately stratify subjects into correct groups (Fig. 5). Furthermore, a Bayes discriminant function model, which enrolled 30 significant metabolites (Supplementary Table S2; Supplementary Figure S5) was established for initial classification screening, which may be helpful for clinical diagnosis. This model has an excellent performance in the multiple classification of clinical samples, with an accuracy of 100% for four groups in training data set, and an accuracy of 100% for NC, 94.12% for HBV, 100% for LC and 76.92% for HCC in the validation data set (Supplementary Table S4).

Based on the detected metabolic changes, the most relevant pathways involved in hepatocarcinogenesis are shown in Supplementary Figure S6, which provides a holistic view of the metabolic features of liver diseases, and gives a better understanding of the potential mechanism of hepatocarcinogenesis.

Glucose 6-phosphate (G6P), which was significantly elevated in HCC (Supplementary Figure S6, Fig. 6), is the start point of two metabolic pathways: glycolysis and the pentose phosphate pathway (PPP). Cancer cells generally exhibit aberrant glycolysis for ATP generation and G6P has been proposed to produce reduced nicotinamide adenine dinucleotide phosphate (NADPH), which supports macromolecular biosynthesis and protects tumor cells from immune injury, or/and ribose as the building blocks for nucleotide synthesis through PPP^34^. NADPH is required for the generation of reduced glutathione (GSH), a non-enzymatic reducing agent that helps to prevent oxidative stress in most cells and to decrease ROS levels^32^. Additionally, marked increases in glutamic acid, cysteine and glycine, which are precursors of GSH, were observed in HCC (Supplementary Figure S6, Fig. 6)^35^. Oxoproline, an important factor in the synthesis and degradation of the GSH pathway was also significantly enhanced in liver diseases compared with NC.

In addition, pyruvate kinase (PK) is a glycolytic enzyme that catalyzes the conversion of phosphoenolpyruvate (PEP) to pyruvate. In tumor cells, the low activity M2 isoform of pyruvate kinase M (PKM2) is predominantly expressed instead of PKM1, which is supposed to facilitate anabolic metabolism by the accumulation of upstream glycolytic intermediates and subsequent shunting of these intermediates into anabolic pathways^23^,^36^. The PKM2 bottleneck causes a reduction in glucose derived metabolites. However, pyruvate, which is required to sustain lactate dehydrogenase activity, was upregulated in HCC, indicating that a large share of pyruvate may derive from sources other than glycolysis. One way to maintain or enhance pyruvate production might be through a proposed alternative glycolytic pathway without ATP generation^22^,^37^. Another way is through the citrate shuttle^31^. Besides, glutamine can provide pyruvate through a conversion of malic acid to pyruvate by the malic enzyme^33^.

Numerous studies have reported the dysregulation of amino acids metabolism in HCC^10^,^11^,^25^,^38^, consistent with these reports, we found that serum levels of alanine, serine, glycine, cysteine, aspartic acid, lysine, methionine, tyrosine, phenylalanine, tryptophan and glutamic acid were dramatically increased in HCC compared with NC (See Supplementary Figure S6). In addition, the ratios of branched-chain amino acids (BCAAs, valine, leucine and isoleuline) to aromatic amino acids (ArAAs, tyrosine, phenylalanine and tryptophan) were lower in HBV, LC and HCC compared with NC (See Supplementary Figure S7), indicating enhanced BCAA catabolism and reduced ArAA breakdown in the liver diseases^39^.

Meanwhile, we observed a gradual up-regulation of the ratio of FFA C16:1 to C16:0 and FFA C18:1 to C18:0 during hepatocarcinogenesis (See Supplementary Figure S7). This is in agreement with the reported results in a mouse model of nonalcoholic steatohepatitis (NASH) and HCC, as a result of significantly increased level of stearoyl-CoA desaturase 1(SCD1), due to the increased demand for lipid synthesis in HCC^40^.

In summary, we performed integrated and comprehensive metabolomics investigations on three liver diseases (HBV, LC and HCC), providing a holistic understanding of the progression of HCC, and identified liver disease-specific potential biomarkers for early diagnosis and clinical staging of liver diseases, with an excellent discriminant performance. Significant metabolic alterations were observed in the progression of HCC. These metabolic adaptations account for the fundamental requirements of tumor cells proliferation: producing sufficient energy and building blocks for macromolecular biosynthesis, and maintaining the redox balance to protect the tumor cells from oxidative stress.

Given the relatively limited cases and analytical platforms used at present, further studies with more samples and multi-analytical techniques are required to confirm these findings. Overall, however, our study indicates that metabolic profiling is a powerful tool to explore the molecular pathogenesis of diseases and identify the potential biomarkers for clinical diagnosis.

## Materials and Methods

### Study subjects

Two hundred and one serum samples were collected from 49 HBV patients, 52 LC patients, 39 HCC patients and 61 healthy subjects (NC) with matched age and genderfrom communities in China with informed consent obtained from all participants. The study was approved by the ethics committee of the National Center of Biomedical Analysis in accordance with the Declaration of Helsinki. The detailed information on the study groups is shown in Supplementary Table S4. All patients were diagnosed by clinical laboratory and imaging evidence. Whole blood samples were collected in the morning before breakfast from all participants by venipuncture into untreated tubes and allowed to clot on ice for a maximum of 2 hours. Serum samples were separated by centrifugation and then stored at −80 °C until analysis.

### Sample Preparation and Analysis

An aliquot of 100 μL from each serum sample was extracted with 500 μL methanol and vortexed vigorously. The solution was spiked with an internal standard (10 μL ribitol solution, 0.2 mg/mL in H_2_O) and vortexed for 30 s. After adding 10 μL of deionized water and vortexed for another 10 s, the samples were placed on a shaker at 100 rpm and 70 °C for 15 min, and subsequently centrifuged at 13000 × g for 15 min. The supernatant was added with 450 μL deionized water and 270 μL chloroform respectively, then placed on a shaker at 200 rpm and 37 °C for 5 min and centrifuged at 4000 × g for 15 min. The final supernatant was dried under a stream of N_2_ gas at 45 °C. The residue was derivatized using a two-step procedure. Firstly, 40 μL methoxyamine hydrochloride (20 mg/mL in pyridine) was added to the residue and shaken at 30 °C for 90 min. The solution was then mixed with 40 μL MSTFA (1% TMCS) and incubated at 37 °C for 30 min. The samples were kept at room temperature for another 120 min, and then stored at 4 °C before injection.

A 1 μL aliquot of the derivatized solution was injected at a split ratio of 1:5 into an Agilent 6890N gas chromatograph coupled with a Pegasus HT time-of-flight mass spectrometer (Leco Corporation, St Joseph, MI, USA). Separation was achieved on a primary column Rsi-5 MS (30 m × 250 μm i.d., 0.25 μm, Agilent J&W Scientific, Folsom, CA, USA) and a secondary column RTX-200 (1.590 m × 180 μm i.d., 0.20 μm, Restek Corp., Belle-fonte, PA, USA) with helium as the carrier gas at a constant flow rate of 1.0 mL/min. The temperature of injection, transfer interface, and ion source was set to 260 °C, 280 °C, and 220 °C, respectively. The GC temperature programming was set at 80 °C for 1 min isothermal heating, then increased to 280 °C with increments of 5 °C/min and held for 10 min. The temperature of secondary column was maintained at 10 °C higher than that of the primary column and the modulator temperature offset was 15 °C higher than that of the secondary column. The solvent acquisition delay was 300 s. The spectrometer was operated in full scan mode (m/z 50–800) with an acquisition rate of 10 spectrum/second. Electron impact ionization at 70 eV was employed with a detector voltage of 1,475 V. Chromatogram acquisition, baseline correction, noise reduction, smoothing, library research and peak area calculation were performed using the ChromaTOF software (Version 4.5, Leco Corp.). Peaks with a signal-to-noise ratio (S/N) greater than 50 were considered and their areas were calculated by the software using unique mass. Peaks with a similarity index (SI) more than 60% were assigned compound names, while those with an SI less than 60% were considered unknown compounds, and were finally verified by available reference compounds.

To assess instrument stability and sample carry over, and to ensure data quality, four different procedures for quality control were conducted during the sample sequence. Firstly, 30 pooled samples prepared by mixing aliquots of all serum samples, were injected once after every 10 sample injections throughout the data acquisition process to evaluate the reproducibility of the metabolic profiling. Secondly, 15 blanks were randomly injected to assess sample carryover. Thirdly, ribitol was chosen as the internal standard to improve the accuracy of the analysis. Finally, 30 duplicate samples (15% of the total samples) were randomly selected and processed at the end of the run to evaluate chromatogram consistency.

### Data processing and statistical Analysis

Smoothing, denoising, peak picking, identification, alignment and normalization of the acquired data were conducted by the Statistical Compare feature of ChromaTOF software. Known interference peaks, such as peaks from column bleed, noise, and derivatization agents, were removed from the dataset. The processed data were imported into SIMCA-P 12.0 software (Umetrics, Umeå, Sweden) for multivariate pattern recognition analysis. PCA was performed to detect outliers and distributions of deferent groups and OPLS-DA was carried out to obtain an overview of the complete data set after mean centering and unit variance (UV) scaling. The RF machine learning algorithm was applied to classify serum samples, and yielded classification accuracy for different groups. The top 30 variables were ranked by mean decrease in accuracy, based on the RF model built on the training data set. These 30 differential metabolites were then imported into SPSS 19.0 software (SPSS Inc., Chicago, IL, USA) for non-conditional logistic stepwise regression (LR) analysis. Two-tailed Welch’s t-tests, one-way analysis of variance (ANOVA) followed by Tukey’s multiple comparison tests, receiver operating characteristic (ROC) analysis, binary logistic regression and Bayes discriminant analysis were also conducted.

## Additional Information

**How to cite this article**: Gao, R. *et al*. Serum Metabolomics to Identify the Liver Disease-Specific Biomarkers for the Progression of Hepatitis to Hepatocellular Carcinoma. *Sci. Rep.* **5**, 18175; doi: 10.1038/srep18175 (2015).

## Supplementary Material

Supplementary Information

## References

[b1] Lanaya H. . EGFR has a tumour-promoting role in liver macrophages during hepatocellular carcinoma formation. Nat. Cell Biol. 16, 972–981 (2014). 25173978 10.1038/ncb3031 PMC4183558

[b2] Dhanasekaran R., Limaye A. & Cabrera R. Hepatocellular carcinoma: current trends in worldwide epidemiology, risk factors, diagnosis, and therapeutics. Hepat. Med. 4, 19–37 (2012). 24367230 10.2147/HMER.S16316 PMC3846594

[b3] Flores A. & Marrero J. A. Emerging trends in hepatocellular carcinoma: focus on diagnosis and therapeutics. Clin. Med. Insights Oncol. 8, 71–76 (2014). 24899827 10.4137/CMO.S9926 PMC4039215

[b4] Parkin D. M. The global health burden of infection-associated cancers in the year 2002. Int. J. Cancer 118, 3030–3044 (2006). 16404738 10.1002/ijc.21731

[b5] Farrell G. C. . Prevention of hepatocellular carcinoma in the Asia-Pacific region: consensus statements. J. Gastroenterol. Hepatol. 25, 657–663 (2010). 20492323 10.1111/j.1440-1746.2009.06167.x

[b6] Yuen M. F., Hou J. L. & Chutaputti A. Hepatocellular carcinoma in the Asia pacific region. J. Gastroenterol. Hepatol. 24, 346–353 (2009). 19220670 10.1111/j.1440-1746.2009.05784.x

[b7] Luo R. H., Zhao Z. X., Zhou X. Y., Gao Z. L. & Yao J. L. Risk factors for primary liver carcinoma in Chinese population. World J. Gastroenterol. 11, 4431–4434 (2005). 16038048 10.3748/wjg.v11.i28.4431 PMC4434676

[b8] Gao H. . Development of T Cells Redirected to Glypican-3 for the Treatment of Hepatocellular Carcinoma. Clin. Cancer Res. 20, 6418–28 (2014). 25320357 10.1158/1078-0432.CCR-14-1170

[b9] Locasale J. W., Vander Heiden M. G. & Cantley L. C. Rewiring of glycolysis in cancer cell metabolism. Cell Cycle 9, 4253–4253 (2014). 21045562 10.4161/cc.9.21.13925

[b10] Beyoglu D. . Tissue metabolomics of hepatocellular carcinoma: tumor energy metabolism and the role of transcriptomic classification. Hepatology 58, 229–238 (2013). 23463346 10.1002/hep.26350 PMC3695036

[b11] Fitian A. I. . Integrated metabolomic profiling of hepatocellular carcinoma in hepatitis C cirrhosis through GC/MS and UPLC/MS-MS. Liver Int. 34, 1428–1444 (2014). 24661807 10.1111/liv.12541 PMC4169337

[b12] Zeng J. . Metabolomics study of hepatocellular carcinoma: discovery and validation of serum potential biomarkers by using capillary electrophoresis-mass spectrometry. J. Proteome Res. 13, 3420–3431 (2014). 24853826 10.1021/pr500390y

[b13] Wang X. . Urine metabolomics analysis for biomarker discovery and detection of jaundice syndrome in patients with liver disease. Mol. Cell Proteomics 11, 370–380 (2012). 22505723 10.1074/mcp.M111.016006 PMC3412968

[b14] Ressom H. W. . Utilization of metabolomics to identify serum biomarkers for hepatocellular carcinoma in patients with liver cirrhosis. Anal. Chim. Acta 743, 90–100 (2012). 22882828 10.1016/j.aca.2012.07.013 PMC3419576

[b15] Gao H. . Application of1H NMR-based metabonomics in the study of metabolic profiling of human hepatocellular carcinoma and liver cirrhosis. Cancer Sci. 100, 782–785 (2009). 19469021 10.1111/j.1349-7006.2009.01086.x PMC11159264

[b16] Nahon P. . Identification of serum proton NMR metabolomic fingerprints associated with hepatocellular carcinoma in patients with alcoholic cirrhosis. Clin. Cancer Res. 18, 6714–6722 (2012). 23136190 10.1158/1078-0432.CCR-12-1099

[b17] Huang Q. . Metabolic characterization of hepatocellular carcinoma using nontargeted tissue metabolomics. Cancer Res. 73, 4992–5002 (2013). 23824744 10.1158/0008-5472.CAN-13-0308

[b18] Tan Y. . Metabolomics study of stepwise hepatocarcinogenesis from the model rats to patients: potential biomarkers effective for small hepatocellular carcinoma diagnosis. Mol. Cell Proteomics 11, M111 010694 (2012). 22084000 10.1074/mcp.M111.010694 PMC3277755

[b19] Chen T. . Random forest in clinical metabolomics for phenotypic discrimination and biomarker selection. Evid. Based Complement. Alternat. Med. 2013, 298183 (2013). 23573122 10.1155/2013/298183 PMC3594909

[b20] Zhang Y., Zhang H., Elizabeth A. & Liu X. Q. Epidemiology of hepatitis B and associated liver diseases in china. Chin. Med. Sci. J. 27, 243–248 (2013). 23294591 10.1016/s1001-9294(13)60009-7

[b21] Xia J. & Wishart D. S. Web-based inference of biological patterns, functions and pathways from metabolomic data using MetaboAnalyst. Nat. Protoc. 6, 743–760 (2011). 21637195 10.1038/nprot.2011.319

[b22] Ward P. S. & Thompson C. B. Metabolic reprogramming: a cancer hallmark even warburg did not anticipate. Cancer cell 21, 297–308 (2012). 22439925 10.1016/j.ccr.2012.02.014 PMC3311998

[b23] Kroemer G. & Pouyssegur J. Tumor cell metabolism: cancer’s Achilles’ heel. Cancer cell 13, 472–482 (2008). 18538731 10.1016/j.ccr.2008.05.005

[b24] Wang X., Zhang A. & Sun H. Power of metabolomics in diagnosis and biomarker discovery of hepatocellular carcinoma. Hepatology 57, 2072–2077 (2013). 23150189 10.1002/hep.26130

[b25] Shao Y. . Development of Urinary Pseudotargeted LC-MS-Based Metabolomics Method and Its Application in Hepatocellular Carcinoma Biomarker Discovery. J. Proteome Res. 14, 906–916 (2015). 25483141 10.1021/pr500973d

[b26] Koppenol W. H., Bounds P. L. & Dang C. V. Otto Warburg’s contributions to current concepts of cancer metabolism. Nat. Rev. Cancer 11, 325–337 (2011). 21508971 10.1038/nrc3038

[b27] Pollard P. J. . Accumulation of Krebs cycle intermediates and over-expression of HIF1alpha in tumours which result from germline FH and SDH mutations. Hum. Mol. Genet. 14, 2231–2239 (2005). 15987702 10.1093/hmg/ddi227

[b28] Shimizu T. . Frequent alteration of the protein synthesis of enzymes for glucose metabolism in hepatocellular carcinomas. J. Gastroenterol. 49, 1324–1332 (2014). 24203292 10.1007/s00535-013-0895-x PMC4156784

[b29] Lee S. . Neuronal apoptosis linked to EglN3 prolyl hydroxylase and familial pheochromocytoma genes: developmental culling and cancer. Cancer cell 8, 155–167 (2005). 16098468 10.1016/j.ccr.2005.06.015

[b30] Balamurugan K. HIF-1 at the crossroads of hypoxia, inflammation, and cancer. Int. J Cancer 10.1002/ijc.29519 (2015). PMC4573780 25784597

[b31] Icard P., Poulain L. & Lincet H. Understanding the central role of citrate in the metabolism of cancer cells. Biochim. Biophys. Acta 1825, 111–116 (2012). 22101401 10.1016/j.bbcan.2011.10.007

[b32] Chen J. Q. & Russo J. Dysregulation of glucose transport, glycolysis, TCA cycle and glutaminolysis by oncogenes and tumor suppressors in cancer cells. Biochim. Biophys. Acta 1826, 370–384 (2012). 22750268 10.1016/j.bbcan.2012.06.004

[b33] Daye D. & Wellen K. E. Metabolic reprogramming in cancer: unraveling the role of glutamine in tumorigenesis. Semin. Cell Dev. Biol. 23, 362–369 (2012). 22349059 10.1016/j.semcdb.2012.02.002

[b34] Cairns R. A., Harris I. S. & Mak T. W. Regulation of cancer cell metabolism. Nat. Rev. Cancer 11, 85–95 (2011). 21258394 10.1038/nrc2981

[b35] Qiu Y. . A distinct metabolic signature of human colorectal cancer with prognostic potential. Clin. Cancer Res. 20, 2136–2146 (2014). 24526730 10.1158/1078-0432.CCR-13-1939 PMC5902798

[b36] Hu J. . Heterogeneity of tumor-induced gene expression changes in the human metabolic network. Nat. Biotechnol. 31, 522–529 (2013). 23604282 10.1038/nbt.2530 PMC3681899

[b37] Vander Heiden M. G. . Evidence for an alternative glycolytic pathway in rapidly proliferating cells. Science 329, 1492–1499 (2010). 20847263 10.1126/science.1188015 PMC3030121

[b38] Chen T. . Serum and Urine Metabolite Profiling Reveals Potential Biomarkers of Human Hepatocellular. Mol. Cell Proteomics 10, M110.004945 (2011). 21518826 10.1074/mcp.M110.004945 PMC3134066

[b39] Chen S. . Pseudotargeted metabolomics method and its application in serum biomarker discovery for hepatocellular carcinoma based on ultra high-performance liquid chromatography/triple quadrupole mass spectrometry. Anal. Chem. 85, 8326–8333 (2013). 23889541 10.1021/ac4016787

[b40] Muir K. . Proteomic and lipidomic signatures of lipid metabolism in NASH-associated hepatocellular carcinoma. Cancer Res. 73, 4722–4731 (2013). 23749645 10.1158/0008-5472.CAN-12-3797 PMC3855016

