# Supplementary material for: Serum Metabolomics to Identify the Liver Disease-Specific Biomarkers for the Progression of Hepatitis to Hepatocellular Carcinoma
Source: Sci Rep. 2015 Dec 10;5:18175. doi: 10.1038/srep18175 (PMC4674760; doi:10.1038/srep18175)
Supplement: Supplementary Information [file srep18175-s1.pdf]

Supplementary Information

for

Serum Metabolomics to Identify the Liver Disease-Specific Biomarkers for the Progression of Hepatitis to Hepatocellular Carcinoma

Rong Gao,[1] Jianhua Cheng,[1] Chunlei Fan[2], Xiaofeng Shi[3], Yuan Cao[4], Bo Sun,[1] Huiguo Ding[2], Chengjin Hu[4], Fangting Dong,[1 *] Xianzhong Yan[1*]

1. National Center of Biomedical Analysis, Beijing 100850, China;
2. Department of Gastroenterology and Hepatology, Beijing You'an Hospital affiliated to Capital Medical University, Beijing 100069, China;
3. Key Laboratory of Molecular Biology for Infectious Diseases, Ministry of Education, China; Department of Infectious Diseases, the Second Affiliated Hospital of Chongqing Medical University, Chongqing 400010, China;
4. Department of Laboratory Medicine, the General Hospital of Jinan Military Command, Jinan 250031, Shandong, China.

Supplementary Table S1. The Pearson correlation coefficient of the randomly selected duplicate samples.

Duplicate sample (DS)	Pearson Correlation Coefficient	Duplicate Sample (DS)	Pearson Correlation Coefficient
**DS-1**	0.998268058	DS-16	0.999686837
**DS-2**	0.999072175	DS-17	0.985738252
**DS-3**	0.999426573	DS-18	0.999634599
**DS-4**	0.999664146	DS-19	0.999440753
**DS-5**	0.999856752	DS-20	0.999939986
**DS-6**	0.999243799	DS-21	0.999047992
**DS-7**	0.999481800	DS-22	0.999790789
**DS-8**	0.999781987	DS-23	0.999958316
**DS-9**	0.998400789	DS-24	0.999389796
**DS-10**	0.997037309	DS-25	0.999946129
**DS-11**	0.999941303	DS-26	0.999935874
**DS-12**	0.999931416	DS-27	0.969182726
**DS-13**	0.999990246	DS-28	0.987997904
**DS-14**	0.999800965	DS-29	0.994257984
**DS-15**	0.999827710	DS-30	0.978366691

Supplementary Table S2. The stepwise discriminant functions variables and classification function coefficients of the four groups.

Metabolites	$C^*_{(CON)}$	$C^*_{(HBV)}$	$C^*_{(LC)}$	$C^*_{(HCC)}$
**Boric acid**	-3.84E-06	8.52E-06	-3.35E-06	-3.84E-06
**Trans-1,2-cyclopropanecarboxylate**	2.60E-06	8.98E-05	4.95E-05	2.60E-06
**Valine**	3.11E-06	-6.35E-06	-8.24E-07	3.11E-06
**Oxalic acid**	7.16E-06	4.00E-05	1.11E-06	7.16E-06
**Isoleucine**	-5.80E-06	-2.43E-06	2.49E-06	-5.80E-06
**3-methyl-2-hydroxybutyric acid**	-1.85E-06	-8.60E-06	-4.01E-06	-1.85E-06
**Ethanolamine**	1.03E-04	-3.62E-04	3.49E-05	1.03E-04
**Leucine**	1.64E-05	-2.37E-05	1.48E-05	1.64E-05
**Succinic acid**	-5.03E-06	-1.32E-05	5.76E-07	-5.03E-06
**Salicylaldehyde**	7.74E-04	6.22E-03	1.09E-03	7.74E-04
**4-Hydroxy-Proline**	-1.93E-06	-4.27E-05	-4.32E-06	-1.93E-06
**Threitol**	3.52E-05	6.37E-05	3.21E-05	3.52E-05
**Methionine**	3.72E-06	-1.57E-05	-6.48E-06	3.72E-06
**Glutamate**	2.33E-06	1.46E-05	3.47E-06	2.33E-06
**Threonic acid**	-3.40E-06	-6.19E-06	-2.07E-07	-3.40E-06
**Glutamate**	5.71E-06	-6.16E-06	2.08E-07	5.71E-06
**Dodecanoic acid**	2.78E-05	-2.07E-04	-5.97E-05	2.78E-05
**Asparagine**	-1.17E-04	1.42E-04	1.44E-04	-1.17E-04
**9H-purine**	-1.65E-05	-2.40E-04	-2.88E-05	-1.65E-05
**Citrulline**	1.61E-04	1.31E-05	-1.99E-06	1.61E-04
**Methionine sulfoxide**	-2.03E-05	2.98E-04	5.30E-05	-2.03E-05
**Fructose**	-8.55E-06	-4.19E-05	-1.71E-05	-8.55E-06
**Glucose**	5.56E-06	-2.60E-05	-1.87E-05	5.56E-06
**Altrose**	-1.56E-05	7.33E-05	-2.17E-05	-1.56E-05
**Arachidic acid**	2.66E-04	-3.50E-04	4.04E-05	2.66E-04
**Methyl -β-D-galactopyranoside**	-1.59E-07	8.61E-07	3.19E-06	-1.59E-07
**Palmitic acid**	2.60E-08	1.02E-05	3.64E-06	2.60E-08

**Noradrenaline**	-7.71E-05	3.31E-04	-1.10E-04	-7.71E-05
**Stearic acid**	-1.05E-06	-9.35E-06	-1.04E-06	-1.05E-06
**Sucrose**	-2.70E-07	1.68E-05	-1.91E-06	-2.70E-07
**Constant**	-1.55E+03	-1.91E+03	-4.68E+02	-2.42E+02

Supplementary Table S3. The Bayes Classification Results of training and validation data sets.

		group	Predicted Group Membership				Total
			NC	HBV	LC	HCC	
**Training data set**	Count	NC	40	0	0	0	40
		HBV	0	32	0	0	32
		LC	0	0	34	0	34
		HCC	0	0	0	26	26
	%	NC	100.00	0. 00	0. 00	0. 00	100.00
		HBV	0. 00	100.00	0. 00	0. 00	100.00
		LC	0. 00	0. 00	100.00	0. 00	100.00
		HCC	0. 00	0. 00	0. 00	100.00	100.00
**Validation data set**	Count	NC	21	0	0	0	21
		HBV	1	16	0	0	17
		LC	0	0	18	0	18
		HCC	0	0	3	10	13
	%	NC	100.00	0. 00	0. 00	0. 00	100.00
		HBV	5.88	94.12	0. 00	0. 00	100.00
		LC	0. 00	0. 00	100.00	0. 00	100.00
		HCC	0. 00	0. 00	23.08	76.92	100.00

Supplementary Table S4. Characteristics of the healthy controls and patients with chronic hepatic diseases and HCC

Characteristics	NC (n=61)	HBV (n=49)	LC (n=52)	HCC (n=39)
Age (years)	40.0 ±9.8 (18~71)	43.9 ±9.5 (20~76)	48.2 ±10.0 (25~72)	54.7 ±8.8 (24~84)
Male (%)	69.2	84.3	78.5	86.2
AFP (range),ng/ml	/	51.85 ±70.2 (1.88~210.2)	211.8 ±387.3 (2.08~1189.3)	233.9 ±430.3 (1.86~1210)
HbsAg	Negative	Positive	Positive	Positive

NC: Healthy control; HBV: Hepatitis B virus; LC: Liver cirrhosis; HCC: Hepatocellular carcinoma; AFP: alpha fetal protein.

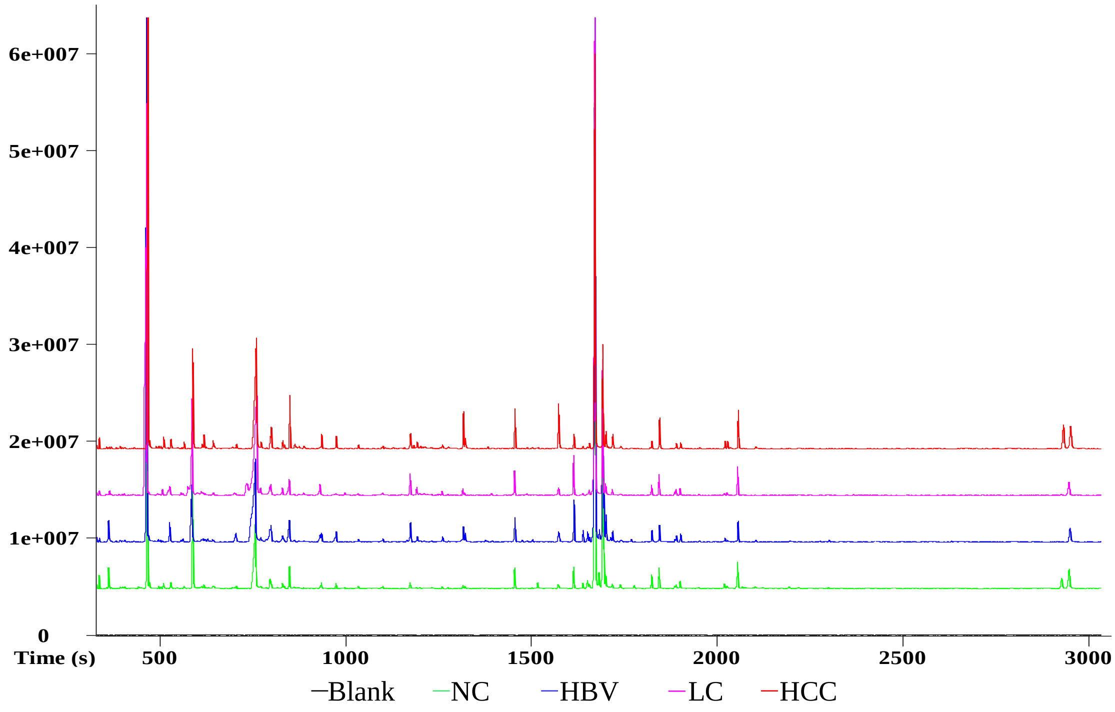

Figure S1. Metabolic profilings of representative serum samples. The GC-TOFMS TIC chromatograms for blank (black line), health control (CN, green line), HBV (blue line), LC (purple line) and HCC (red line). X axis and Y axis represent the retention time (s) and relative peak intensity, respectively.

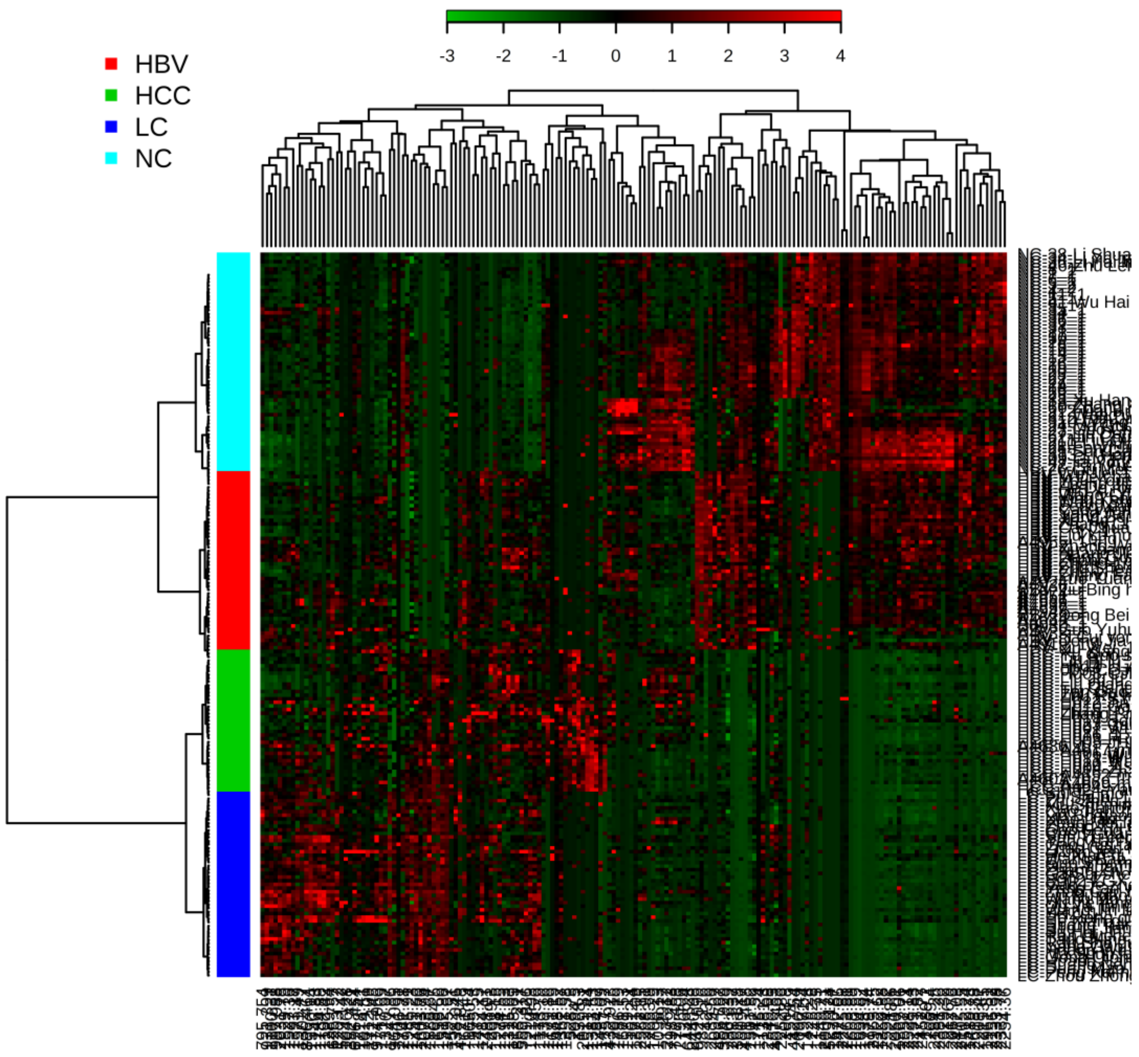

Figure S2. Heat map based on the Pearson distance measure and the Ward cluster algorithm, providing intuitive visualization of the metabolic remodeling in disease groups compared to the control group. Red color indicates a high level of metabolites and green color indicates a low level of metabolites, while black color means an equal level in groups.

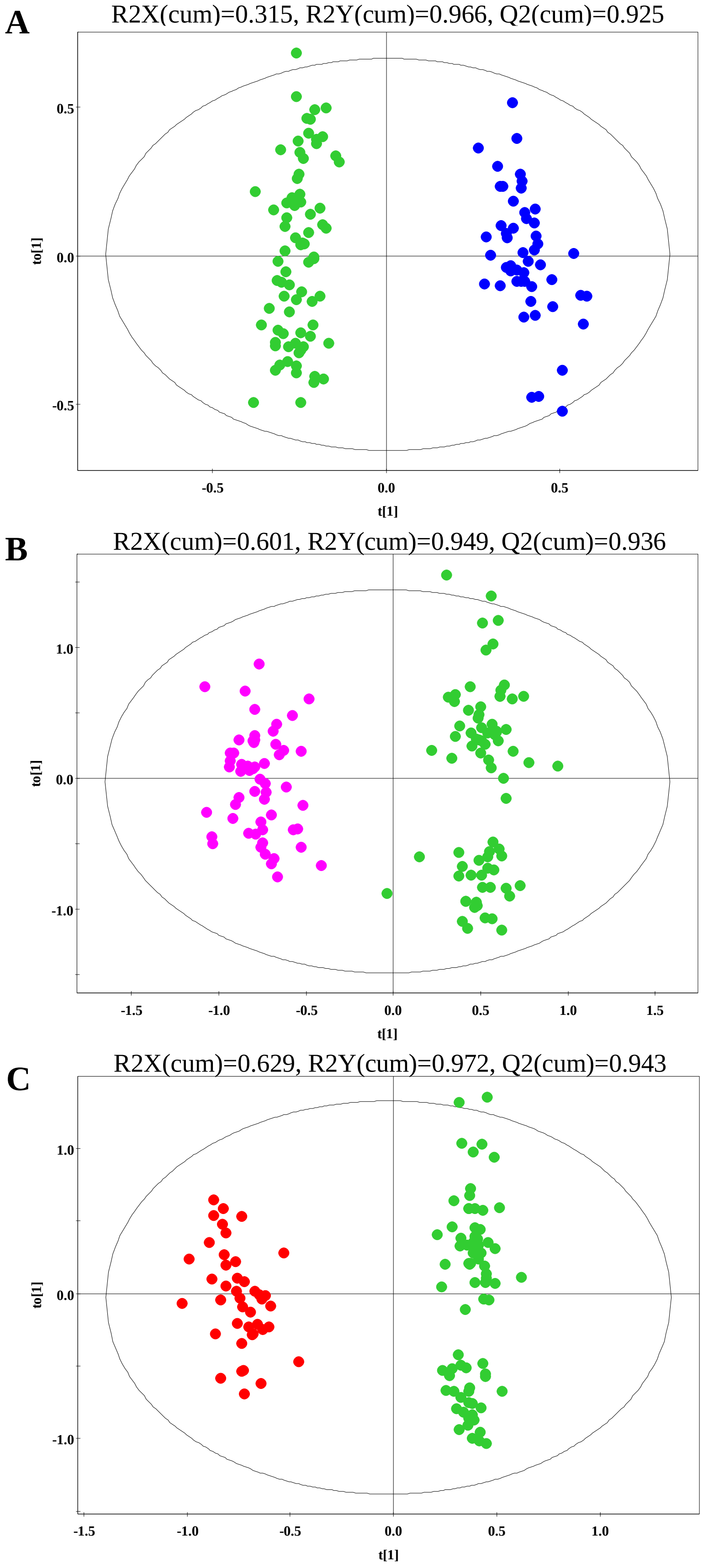

Figure S3. OPLS-DA scores plots for serum samples of (A) patients with HBV (●) and NC (●), (B) patients with LC (●) and NC (●), and (C) patients with HCC (●) and NC (●).

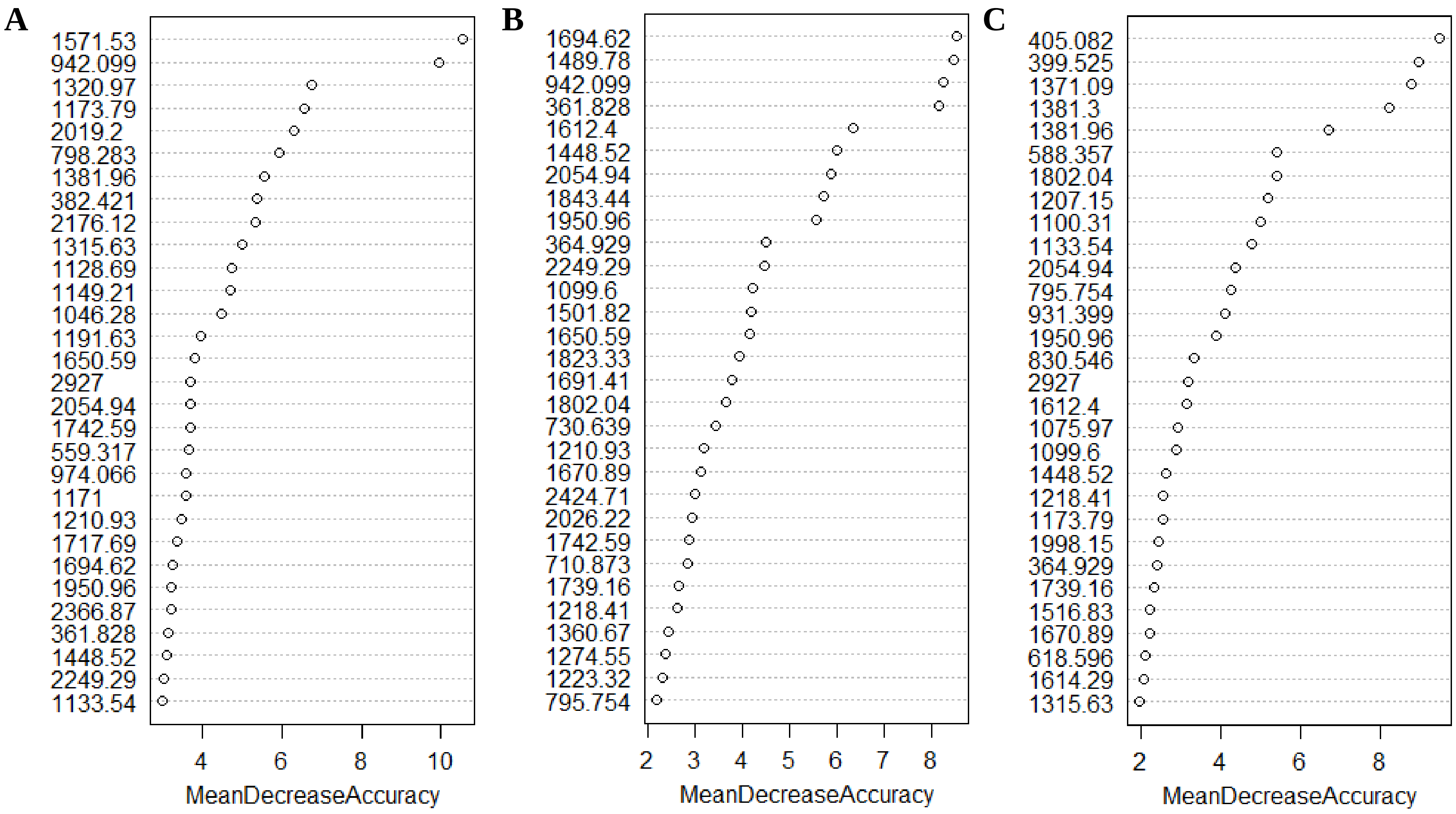

	Predicted HBV	Predicted NC
True HBV	32	0
True NC	0	40

	Predicted LC	Predicted HBV
True LC	34	0
True HBV	0	32

	Predicted HCC	Predicted LC
True HCC	26	0
True LC	0	34

Figure S4. The top 30 ranked differential metabolites according to the mean decrease accuracy (MDA) and performance of Random Forest (RFs) models discriminating (A) HBV from NC, (B) LC from HBV and (C) HCC from LC based on training data set serum.

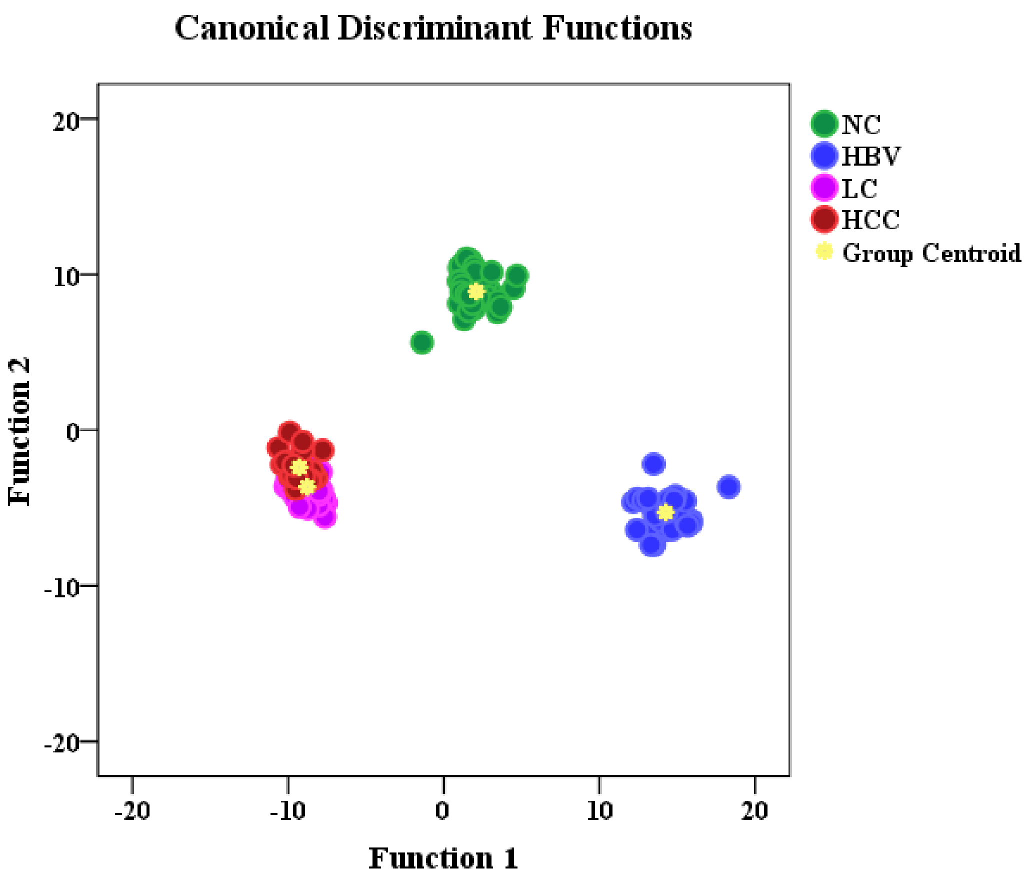

Figure S5. The discriminant function model based on Bayes' Rule by stepwise discriminant analysis.

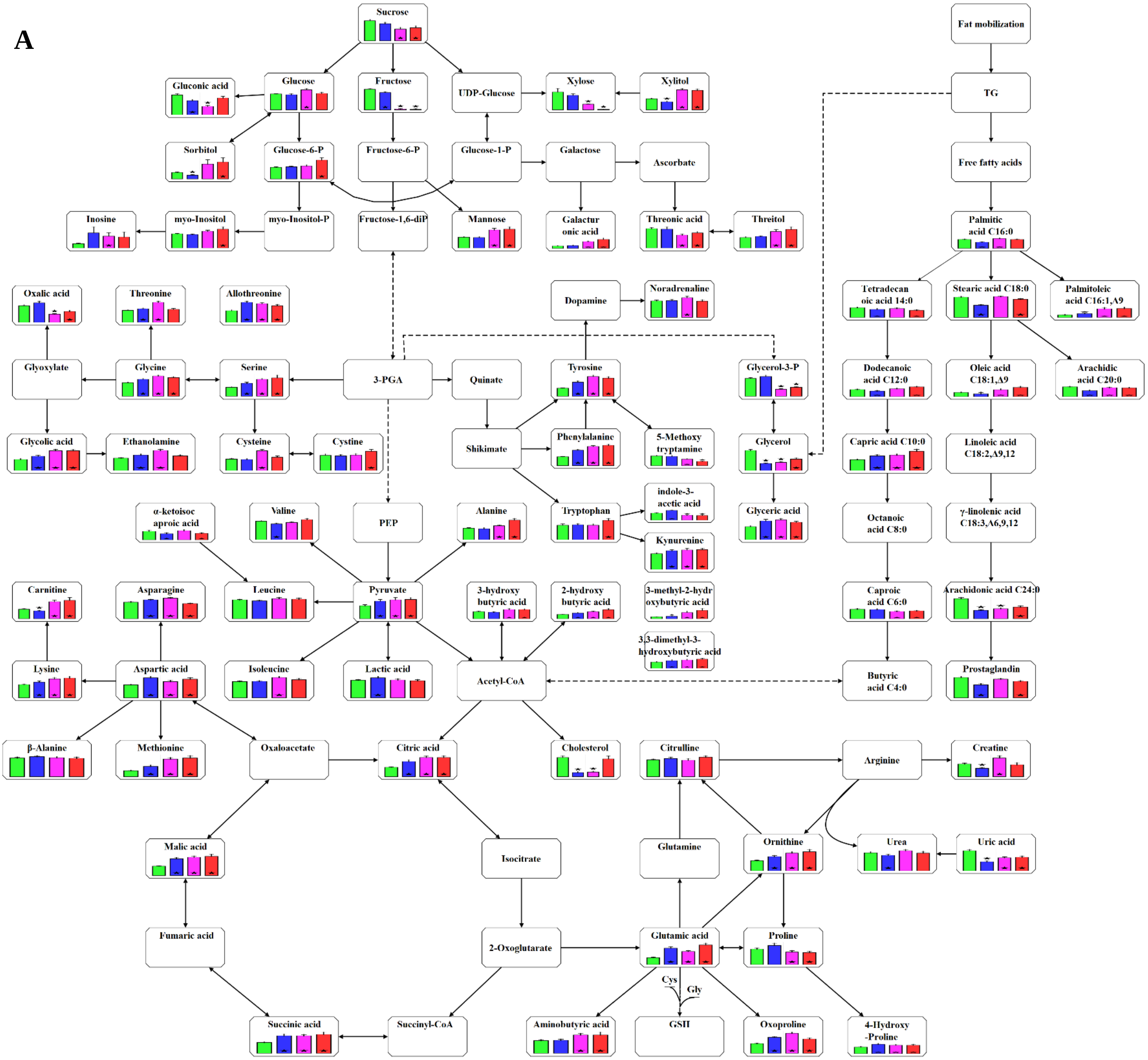

Figure S6. Metabolic network of the detected metabolites in this study. The normalized contents of each metabolite in NC (green bar chart), HBV (blue bar chart), LC (purple bar chart) and HCC (red bar chart) are shown under the chemical name.

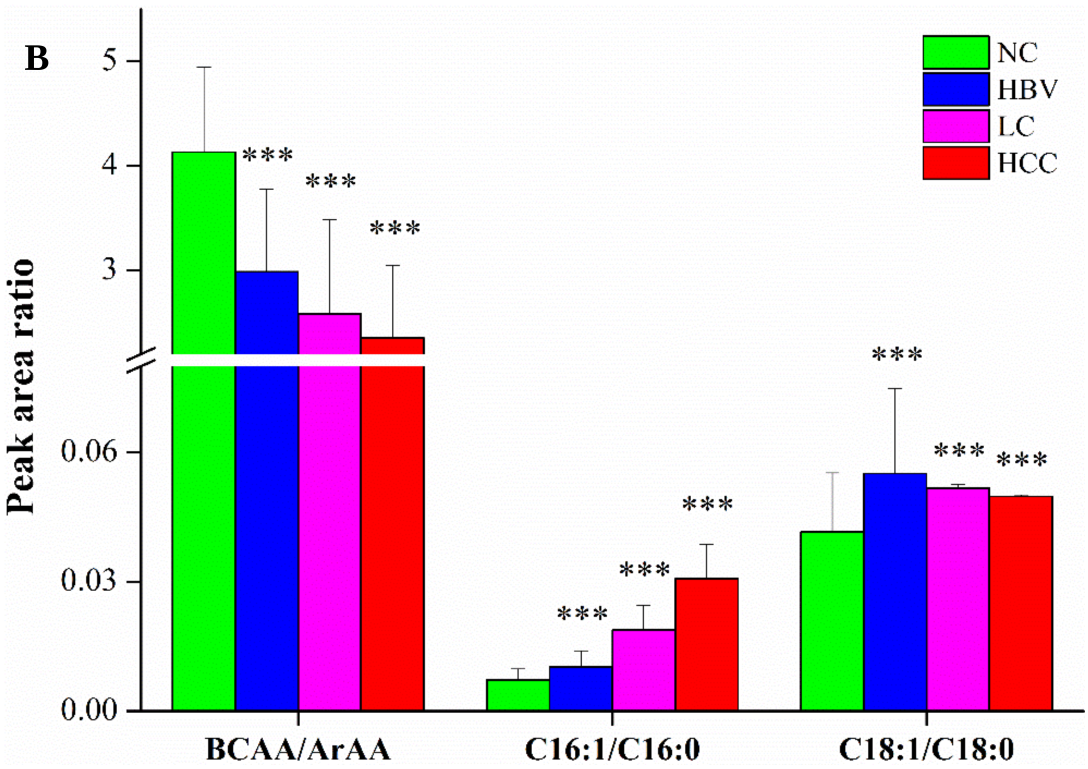

Figure S7. The ratio of BCAA (leucine, isoleucine, and valine) to ArAA (tyrosine, phenylalanine and tryptophan), the ratio of FFA C16:1 to FFA C16:0 and FFA C18:1 to FFA C18:0.
